# The Effect of Deoxycholic Acid on Chitosan-Enabled Matrices for Tissue Scaffolding and Injectable Nanogels

**DOI:** 10.3390/gels8060358

**Published:** 2022-06-07

**Authors:** Bozica Kovacevic, Corina Mihaela Ionescu, Melissa Jones, Susbin Raj Wagle, Michael Lewkowicz, Maja Đanić, Momir Mikov, Armin Mooranian, Hani Al-Salami

**Affiliations:** 1 The Biotechnology and Drug Development Research Laboratory, Curtin Medical School and Curtin Health Innovation Research Institute, Curtin University, Perth, WA 6102, Australia; bozica.kovacevic@postgrad.curtin.edu.au (B.K.); c.ionescu@postgrad.curtin.edu.au (C.M.I.); melissa.a.jones@postgrad.curtin.edu.au (M.J.); susbinraj.wagle@postgrad.curtin.edu.au (S.R.W.); mikhael.lewkowicz@graduate.curtin.edu.au (M.L.); 2 Hearing Therapeutics Department, Ear Science Institute Australia, Queen Elizabeth II Medical Centre, Perth, WA 6009, Australia; 3 Department of Pharmacology, Toxicology and Clinical Pharmacology, Faculty of Medicine, University of Novi Sad, 21101 Novi Sad, Serbia; maja.djanic@mf.uns.ac.rs (M.Đ.); momir.mikov@mf.uns.ac.rs (M.M.)

**Keywords:** bile acid, deoxycholic acid, chitosan, hydrogel, nanogel, biomaterial, bioenergetics

## Abstract

The pathophysiology of a multitude of diseases is influenced by bioenergetic dysfunction. Healthy mitochondria are presented as essential for the regulation and function of multiple cell types, including the cells of relevance for this research: pancreatic beta cells, muscle cells, and liver cells. Hence, effects of hydrogels (particularly nanogels) on bioenergetics needs to be taken into account when designing optimum delivery matrices. Several polymers have been suggested for use in hydrogels and nanogels, with focus on chitosan due to its range of beneficial properties. Bile acids have emerged as beneficial excipients, including deoxycholic acid, which can increase membrane permeability of cells. Nanogels were manufactured containing various concentrations of chitosan and deoxycholic acid in addition to the staple sodium alginate. Nanogels then underwent an array of analysis including rheological studies and in vitro cell work assessing viability, hypoxia, and the bioenergetic profiles. Overall, deoxycholic acid showed enhanced gel strength although this resulted in slightly lower cell viability and impacted bioenergetic profiles. Results from this study showed the benefits of deoxycholic acid; however, this was found to be less suitable for cell delivery matrices and is perhaps more beneficial for drug-delivery systems.

## 1. Introduction

Mitochondria are capable of transforming caloric intake into molecular energy adenosine triphosphate (ATP), metabolically active compounds, thermal energy, and oxidants. They are highly responsive to their environment and oxidative stress. Therefore, it is no surprise that the pathophysiology of many diseases, including diabetes, hearing loss, obesity, cancer, neurodegenerative and cardiovascular diseases, are underlined by bioenergetic dysfunction. Furthermore, mitochondria are essential in modulating reactive oxygen species (ROS) and controlling cellular apoptosis. However, they are sensitive to increased levels of ROS and reactive nitrogen species (RNS) in addition to calcium, endoplasmic reticulum stress, and the presence of modified proteins [1,2,3,4]. Damaged mitochondria can cause significant damage to the host cell, releasing an inappropriate amount of ROS and calcium. This in turn damages the surrounding mitochondria by furthering calcium overload and ROS damage [1].

Muscle cells form highly energy-demanding tissue, with the molecular energy in the form of ATP needed to support its function, mainly for actin–myosin contractions. Skeletal muscle defects are linked to reduced mitochondrial calcium uptake and oxygen consumption rate [5]. Reduced oxidative phosphorylation (OXPHOS) leads to changes in cellular bioenergetics, shifting metabolism to the fatty acid pathway [6].

Mitochondrial disorders underline some of the most common liver disorders, including non-alcoholic fatty liver disease, non-alcoholic steatohepatitis, fibrosis, and steatosis [7]. Hepatocytes with increased mitochondrial matrix ROS are susceptible to mitochondrial DNA damage. ROS activate signalling factors (adenosine monophosphate-activated protein kinase (AMPK) and c-Jun N-terminal kinase (JNK)) involved in disease progression [7,8,9,10].

Mitochondria are essential for insulin secretion from pancreatic beta cells. Beta cells sense glucose concentration by coupling glycolysis to OXPHOS and the citric acid cycle to produce more ATP. This action increases the ATP-to-ADP ratio, leading to inhibition of ATP-inhibiting potassium channels, increased calcium influx, and subsequent exocytosis of insulin vesicles [11]. Mitochondrial disorders in OXPHOS and the citric acid cycle lead to a loss of sensitivity in glucose-stimulated insulin secretion. This is associated with insulin resistance and type 2 diabetes [12].

Therefore, healthy mitochondria are a prerequisite for any tissue function and survival, especially ones encapsulated or embedded in an artificial, extracellular matrix mimicking the environment of tissue replacement devices. Conditions within these vehicles are hypoxic, with uneven oxygen and glucose supply, requiring robust bioenergetics metabolism for tissue to preserve its functionality and viability [13,14,15].

Various polymers are used in cell delivery systems, including nanogels and nanogels, with natural origins, such as chitosan. Chitosan is a cationic linear polymer comprised of two subunits—D glucosamine and N-acetyl-D glucosamine, connected by a glycosidic bond [16]. It is biocompatible, biodegradable, and mucoadhesive, with antioxidant, antimicrobial, and immune-modulating properties, making it a prime candidate for pharmaceutical and cosmetic applications [17]. Chitosan has been used as a controlled drug-delivery system, including both time-controlled and response-stimulated release systems [18]. It has been used as a vehicle for a broad range of pharmaceuticals, including antibiotic, anaesthetic, hypotensive, and chemotherapeutic drugs [19]. Some therapeutical forms include wound dressings [20] and self-healing injectable nanogels [21]. Zhou et al. used a novel crosslinking model to 3D print chitosan scaffold suitable for tissue engineering [22]. By utilising different crosslinking agents, including other polymers, to obtain interpenetrating (IPN) and semi-interpenetrating polymer networks (semi-IPN), it is possible to modulate the self-healing, degradation, and mechanical properties of chitosan hydro/nano-gels [18].

Bile acids are emerging excipients in different pharmacologic and tissue engineering systems [23]. Naturally occurring secondary bile acid, namely deoxycholic acid (DCA), was found to exert positive and stabilising effects on nanogel capsules for oral delivery of a hydrophobic drug [24]. Majimbi et al. observed greater bioavailability of cannabinol when administered in the form of DCA-enhanced microcapsules [25]. Li et al. designed DCA-based micelles for targeted drug delivery of an antitumor agent [26]. The same bile acid was used as part of micelles for drug and gene for cancer therapy [27]. Furthermore, DCA was used as part of poly-l-lysine nanoparticles for the pH-sensitive release of curcumin [28].

DCA is known to enhance membrane permeability and interfere with normal gap junction function [29]. Higher than physiological concentrations can cause local low-grade inflammation and aggravate intestinal tumorigenesis, induce dysbiosis, and disrupt bile acid metabolism in the liver [30,31,32]. Therefore, formulation optimisation is crucial to avoid DCA-associated risks while maintaining the best-performing environment for cells.

Different cells require individual micro and macro environments with a variety of specific variables, so creating one universal cell delivery system would be incredibly challenging, hence our reasoning for investigating novel DCA-nanogels on three cell lines (muscle, liver, and pancreatic beta cells) to observe the effects on different tissues. Moreover, we examined different concentrations within the complex nanogel to better understand DCA’s effect on cellular viability and mitochondrial metabolism. Five different formulations were made, labelled F1 to F5. F1 is made from water-soluble gel and sodium alginate (SA), while F2 contains chitosan in addition to water-soluble gel and SA. Formulations F3, F4, and F5 are made from water-soluble gel, SA, chitosan, and DCA in different concentrations, with F3 having the lowest and F5 containing the highest DCA concentration.

## 2. Results and Discussion

**Figure 1:**
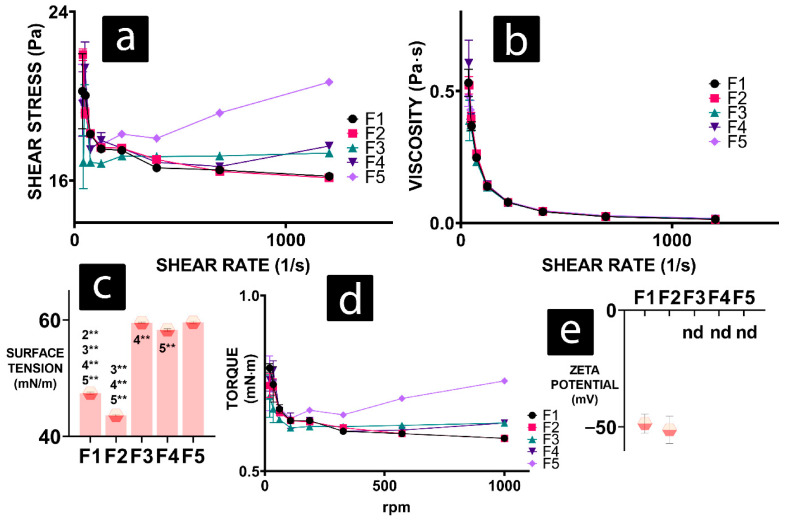
Rheology results of F1 to F5 formulations presented as values of (**a**) shear stress, (**b**) viscosity, (**c**) surface tension, (**d**) torque, and (**e**) zeta potential. *p* < 0.01 (*) or *p* < 0.05 (**).

Formulations with DCA have increased shear stress on higher shear rates, while F1 and F2 show similar, lower values (Figure 1a). Viscosity values for all formulations show a similar trend of decreasing viscosity with increasing shear rate (Figure 1b). Surface tension (Figure 1c) of formulations with DCA show a significant increase compared to DCA-free formulations (F1 and F2). The addition of chitosan (F2) to the gel-alginate matrix (F1) decreases surface tension. Torque results follow the trend of shear stress, with F5 exhibiting the highest torque at high rpm (Figure 1d). Similar to shear stress, DCA-free formulations F1 and F2 follow the same trend and exhibit the lowest values at high rpm among all gels. Zeta potential was successfully measured only for DCA-free gels, and they do not statistically significantly differ from one another (Figure 1e).

All formulations exhibited shear-thinning/thixtropric/non-Nitonion behaviour (Figure 1a,b) characterised by viscous flow under shear stress [33]. Shear-thinning gels are found to be cytoprotective during the injection process and manufacturing of 3D-printed scaffolds. They are preferred to shear thickening gels or Newtonian fluids in cell delivery applications [34]. Shear stress and torque values (Figure 1a,d) show elastic tendencies for F5, a formulation with the highest percentage of DCA. DCA-free formulations (F1 and F2) exhibit the least shear or torque stress (Figure 1a,d). This suggests that DCA is integral in increasing the strength of inter-gel bonds, mainly between chitosan and sodium alginate. This is further observed with surface tension (Figure 1c). DCA-free gels have a smaller surface tension compared to formulations with DCA, suggesting that there are higher energy bonds in the presence of DCA.

**Figure 2:**
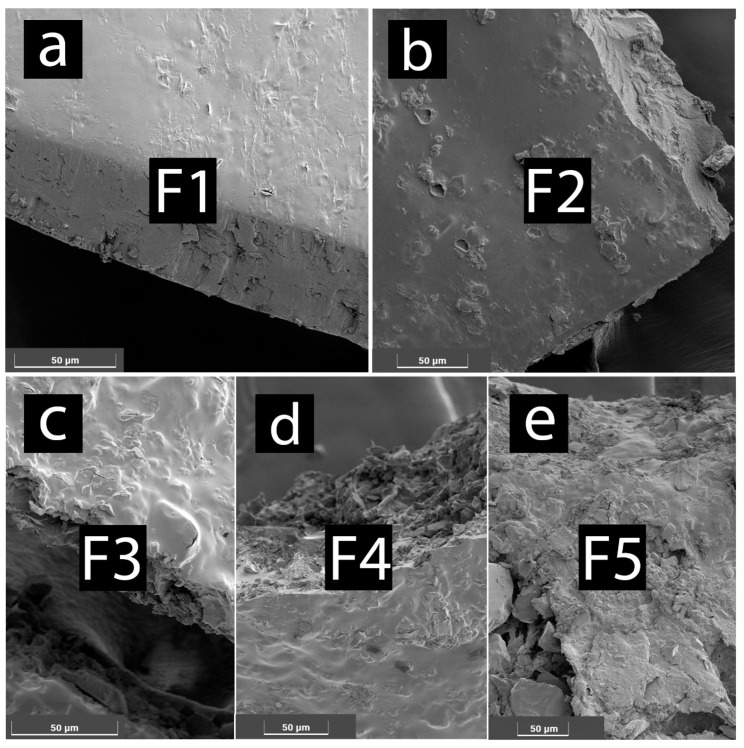
SEM images showing surface morphology of freeze-dried nanogels: (**a**) F1, (**b**) F2, (**c**) F3, (**d**) F4, and (**e**) F5.

SEM images of freeze-dried samples show overall similar surface topography (Figure 2). F1 formulations show the smoothest morphology, (Figure 2a) while addition of chitosan (Figure 2b) results in uneven surface topography. Presence of DCA in gel matrix leads to irregular surface morphology as well as different inner morphology (Figure 2c–e). Surface morphology may be linked to presence of DCA in nanogel matrix, as irregularities in morphology seem the most prevalent in F5 (Figure 2e).

**Figure 3:**
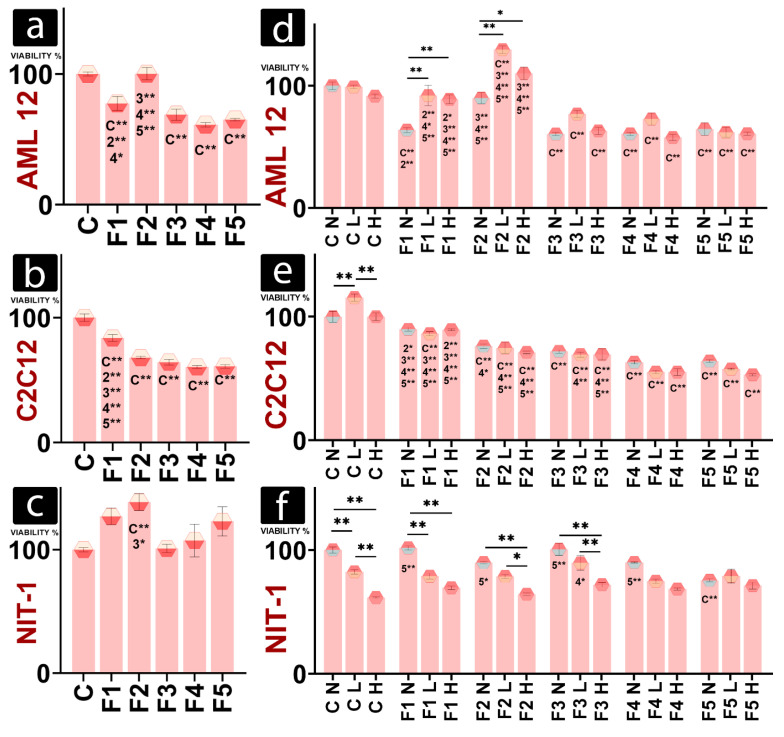
Viability of AML12 (**a**), C2C12 (**b**), and NIT-1 (**c**) under normal conditions. Viability of hepatic (**d**), muscle (**e**), and pancreatic beta cells (**f**) under different hypoxic conditions, where N, non-hypoxic control environment; L, lower hypoxic environment; and H, higher hypoxic environment. *p* < 0.01 (*) or *p* < 0.05 (**).

Viability of hepatic, AML-12 cells in gels was preserved only at F2 compared to the control (C) (Figure 3a). All the other formulations had significantly lower viability in normal conditions after 24 h incubation, compared with control. The beneficial effect of chitosan on liver tissue is documented in the literature, which can explain high viability in the presence of chitosan enriched F2, as chitosan had a protective effect against CCl_4_-induced hepatic fibrosis [35]. Furthermore, Lee et al. constructed a bio-artificial liver chip from chitosan microfibers [36]. The viability of C2C12 decreased compared to gel-free control for all formulations, but it showed no significant difference between different DCA concentrations (F3, F4, and F5) and the chitosan-enhanced formulation (F2) (Figure 3b). NIT-1 cells showed only a significant difference between the gel-free control and F2, with F2 increasing viability (Figure 3c).

Different hypoxic conditions did not significantly affect the viability of gel-free hepatic cells or the viability of cells in the presence of DCA-enriched gels (Figure 3d). Viabilities of AML-12 showed increased values under hypoxia than in non-hypoxic conditions for F1 and F2. Within different hypoxic conditions, values follow a similar trend to data obtained in normal conditions, with increased viability for F2 and decreased for F3, F4, and F5 (Figure 3d), suggesting the positive effect of chitosan.

Gel-free muscle cells were the only ones to respond to hypoxia, with increased viability in lower hypoxia (Figure 3e). This is in accordance with the study by Sakushima et al., who found that slight hypoxia induces proteins needed for muscle hypertrophy and differentiation [37]. Cells in the presence of gels did not respond to hypoxia and showed no significant change in viability under hypoxia. Within the same hypoxic conditions, muscle cells followed the same declining trend as those in normal conditions, with a significant decrease in viability compared to control cells.

Control NIT-1 cells responded to hypoxic conditions in accordance with the severity of hypoxia (Figure 3f). The viability decreased with the increase of hypoxia. This trend was also observed with F1, F2, and F3, while cells in the presence of F4 and F5 had no significant changes in viability regardless of the environment (Figure 3f). Higher viability in the presence of F2, observed in normal conditions (Figure 3c), was not present under the hypoxic or the control environment (Figure 3f). This may suggest that high concentrations of DCA can induce hypoxic conditions or impair aerobic metabolism in NIT-1 cells.

**Figure 4:**
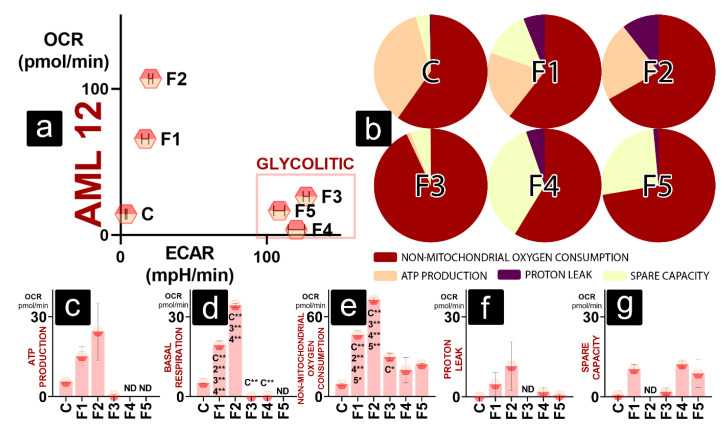
Bioenergetics of AML 12 cells showing (**a**) metabolic profile in normal conditions; (**b**) maximal OCR established as 100% and the oxygen consumption allotted between non-mitochondrial OCR, ATP-linked OCR, proton-leak-linked OCR, and spare-capacity-linked OCR; OCR values for (**c**) ATP production, (**d**) basal respiration, (**e**) non-mitochondrial oxygen consumption, (**f**) proton leak, and (**g**) spare capacity. *p* < 0.01 (*) or *p* < 0.05 (**).

Hepatic cells in a normal environment showed an increase in oxygen consumption in F1 and F2 (Figure 4a). For DCA gels (F3, F4, F5), an increase in extracellular acidification rate (ECAR) is prominent without an increase in OCR, suggesting a shift to glycolic metabolism (Figure 4a). In Figure 3b, the maximal oxygen consumption rate (OCR) is established as 100% to visually show allocated OCR for different functions within cells in the presence of different gels. Gel-free cells show the highest percentage of OCR allocated to ATP production, with minimal proton leak. F1 has a similar % of non-mitochondrial OCR (NM OCR) with decreased ATP production but increased spare capacity and proton leak. F2 has similar ATP production to F1, but spare capacity is negligible, and proton leak is further increased. F3 shows a substantial increase in non-mitochondrial OCR, while ATP production is deficient. Spare capacity is preserved similarly to gel-free control, and proton leak is insignificant.

In contrast to F3, the fraction of NM OCR of F4 is similar to one of the control free cells (C). F5 shows a higher percentage of NM OCR to F4, but, similar to F4, its OCR is mainly allocated for NM OCR and spare capacity (Figure 4b). ATP production is not observed; however, the spare capacity fraction for F4 is several-fold higher than the same fraction of gel-free cells.

ATP production for hepatic cells did not significantly differ amongst groups due to the high variability of data. Cells in the presence of F4 and F5 have no detectable ATP-production-linked OCR (Figure 4c). However, OCR values for basal respiration follow the trend seen in Figure 3a, with F1 and F2 showing a significant increase in OCR compared to the control, and F3 and F4 had a significant decrease compared to the same variable. Basal respiration of cells in F5 was of non-detectable levels (Figure 4d). Figure 4e shows that an increase in NM OCR features in an increase of total OCR for F1 and F2, which is significantly higher than the control levels. F3 also shows a significant increase in non-mitochondrial OCR, while levels found in F4 and F5 were not significantly different to levels of the control (Figure 4e). While values for proton leak and spare-capacity-linked OCR were detected in most groups, they showed no statistical significance compared to the control cells or between different gels groups (Figure 4f,g).

**Figure 5:**
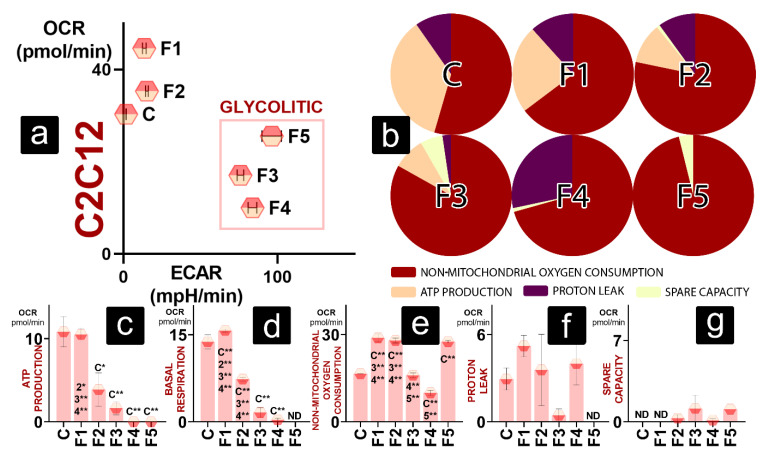
Bioenergetics of C2C12 cells showing (**a**) metabolic profile in normal conditions; (**b**) maximal OCR established as 100% and the oxygen consumption allotted between non-mitochondrial OCR, ATP-linked OCR, proton-leak-linked OCR, and spare-capacity-linked OCR; OCR values for (**c**) ATP production, (**d**) basal respiration, (**e**) non-mitochondrial oxygen consumption, (**f**) proton leak, and (**g**) spare capacity. *p* < 0.01 (*) or *p* < 0.05 (**).

Muscle cells in a normal environment reflect a trend present with hepatic cells—F1 and F2 show an increase in total OCR, while F3, F4, and F5 have increased ECAR and lower total OCR than the control (Figure 5a). However, OCR allocation is different between C2C12 and AML 12 cells. Control cells allocate most of the OCR for non-mitochondrial OCR, ATP production, and proton leak (Figure 5b). F2 and F3 follow that pattern with an increase in NM OCR and decreased ATP production. F3 presents a higher fraction of NM OCR than the control and DCA-free formulations, with smaller ATP production and proton leak. The spare capacity fraction of F3 is more considerable than its proton leak fraction. Maximal OCR allocation of F4 shows high NM OCR and the highest fraction of proton leak of all the formulations. Mitochondrial OCR in F5 is modest and reserved just for spare-capacity-linked OCR, while most oxygen is linked with NM OCR (Figure 5b).

As observed in Figure 5c, only F1 retained ATP production comparable to that of the control. All the other groups (F2, F3, F4, and F5) had significantly decreased ATP-production-linked OCR. OCR linked with basal respiration follows a similar trend (Figure 5d). NM OCR is higher for F1, F2, and F5 than the control, while F3 has comparable and F4 lower values (Figure 5e). Similar to AML 12, values for proton-leak- and spare-capacity-linked OCR were detected for most groups, but no statistical difference was found (Figure 5f,g).

**Figure 6:**
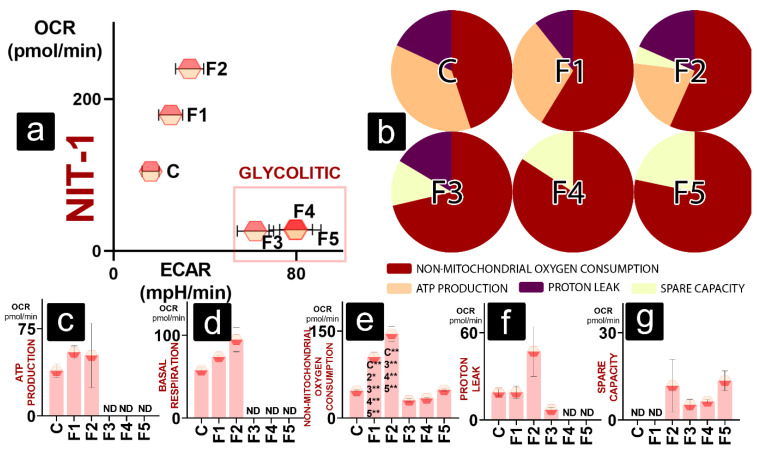
Bioenergetics of NIT-1 cells showing (**a**) metabolic profile in normal conditions; (**b**) maximal OCR established as 100% and the oxygen consumption allotted between non-mitochondrial OCR, ATP-linked OCR, proton-leak-linked OCR, and spare-capacity-linked OCR; OCR values for (**c**) ATP production, (**d**) basal respiration, (**e**) non-mitochondrial oxygen consumption, (**f**) proton leak, and (**g**) spare capacity. *p* < 0.01 (*) or *p* < 0.05 (**).

Pancreatic beta cells show an even more significant disparity between test formulations and the control; F1 and F2 with high total oxygen consumption; and F3, F4, and F5 with a lower total OCR and higher ECAR (Figure 6a). Gel-free NIT-1 cells (Figure 1b) have a similar OCR for NM OCR and ATP production. The rest of the maximal OCR is utilised for proton leak. F1 has slightly increased NM OCR compared to the control and decreased proton leak, while ATP production OCR fraction seems to be retained. ATP production and proton leak consume similar percentages of oxygen in F2, with ATP production being lower than in control and F2. NM OCR is the dominant oxygen consumer for F3, F4, and F5. Next to non-mitochondrial OCR, oxygen is used for proton leak (F3) and spare capacity (F3, F4, and F5) (Figure 6b).

ATP-production-linked OCR was detected only for DCA-free cells (C, F1, and F2), with no statistical difference between groups (Figure 6c). A similar trend is observed with basal respiration (Figure 5d). NM OCR (Figure 5e) was the highest for cells in the presence of DCA-free gels (F1 and F2), which was significantly higher than in control and cells exposed to DCA gels (F3, F4, and F5). This illustrates that most of the increase in total OCR for F1 and F2 (Figure 6a) comes from increased NM OCR rather than mitochondrial respiration. Proton-leak- and spare-capacity-linked OCR showed no significant differences between groups (Figure 6f,g)

All three cell lines follow a similar bioenergetic pattern in the presence of DCA, shifting to anaerobic metabolism, with decreased ATP-linked OCR and increased percentage of spare-capacity-linked OCR and non-mitochondrial OCR.

Cells have enhanced glycolysis in a limited oxygen supply or if OXPHOS is impaired [38]. OXPHOS impairment is visible in the absence of ATP-linked OCR in cells exposed to DCA. ATP production in cells is driven by ATP synthetase, complex V of electron transport chain (ETC). In the seahorse Mito stress assay, ATP synthetase is inhibited by oligomycin, and ATP production OCR is calculated as a difference in OCR before and after oligomycin injection. Oligomycin injection does not seem to be effective in most cells in the presence of DCA, suggesting prior ATP synthetase inhibition.

The main reasons behind compromised ATP production are damaged ETC, low substrate availability, and low ATP demand [3], meaning that DCA can exacerbate one or more of those conditions. Damaged ETC is characterised by high proton leak and low spare capacity. The injection of uncoupling agent (p-(trifluoromethoxy) phenylhydrazone FCCP) depletes mitochondrial membrane potential, allowing for uninhibited electron flow through ETC. The addition of FCCP estimates maximal OCR without dependence on ATP/ADP transport [39], as ATP/ADP translocator is dependent on proton gradient [40]. Therefore, spare capacity is the oxygen consumption available to cells during stress and increased ATP demand [39]. However, investigated cell types show no significant difference between proton-leak- and spare-capacity-linked OCR. This may suggest that DCA does not compromise ETC integrity.

Rheology studies (Figure 1) suggest increased bond strength within DCA gels, leading to thicker, more viscous gels. It is possible that oxygen and nutrients were unable to reach cells embedded in the DCA gel, leading to hypoxia. Hypoxic conditions lead to the stabilisation of hypoxia inducing factor HIF1 α. Hypoxia-inducing properties of CoCl_2_, used in an assay to obtain results presented in Figure 3, rely on the exact HIF1 α mechanism [41]. Cells in non-hypoxic conditions in the presence of DCA mostly had unchanged viability to the cells treated to CoCl_2_ (Figure 3d–f), so it can be argued that they were already hypoxic, with their HIF1 α stabilised prior to the CoCl_2_ treatment. HIF1 α leads to the increased expression of enzymes needed for glycolysis, including glyceraldehyde-3-phosphate dehydrogenase [42,43]. HIF1 α also modulates pH in cells by enhancing lactic acid production [44]. Hypoxia and HIF1 α regulate the expression of ATPase Inhibitory Factor 1 (IF1) [45]. IF1 has pH-regulated binding to ATP synthetase, preferring the acidic environment available with an increase in glycolysis [46]. IF1 expression leads to the upregulation of aerobic glycolysis and inhibits ATP production in cells by inhibiting ATP synthase [47]. This may be the pathway responsible for glycolytic metabolism shift and absence of significant decrease in APT-production-linked OCR in cells in the presence of DCA gels, as IF1 mimics the effects of oligomycin. Furthermore, IF1-mediated inhibition of ATP synthase leads to mitochondrial hyperpolarisation and the production of ROS [48]. However, in large amounts, ROS lead to lipid peroxidation, DNA damage, protein oxidation, ATP-synthase inhibition, mitochondria damage, and cell death [49]. This is concurrent with Yerushalmi et al., who found that glycochenodeoxycholic acid and hydrophobic bile acid, such as DCA, induces ROS generation and mitochondrial permeability transition in hepatocytes [50]. Excessive levels of hydrophobic bile acid stimulate mitochondria to release ROS, and high cytoplasmic ROS induces Ca^2+^ to exit the endoplasmic reticulum to the cytosol, where it further stimulates mitochondria to produce ROS [51]. This may be why NM OCR did not decrease together with OXPHOS in the presence of DCA (F3, F4, and F5) but increased in most cells (Figure 4e, Figure 5e and Figure 6e).

**Figure 7:**
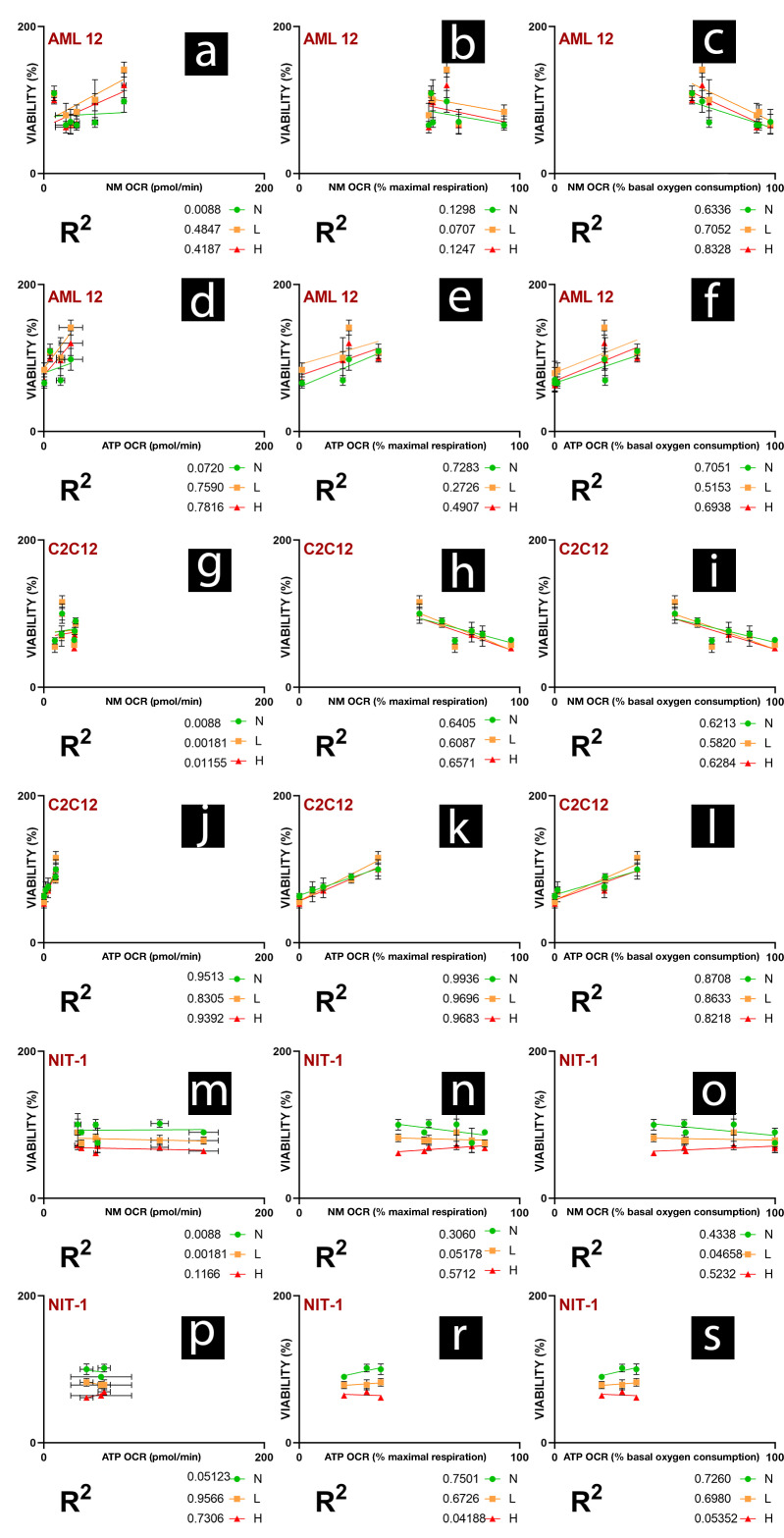
Relationships between bioenergetics in normoxic conditions and viability under normal, low, and high hypoxia. NM OCR, non-mitochondrial-linked OCR; ATP OCR, ATP-production-linked OCR. % maximal respiration—parameter is expressed as a % of maximal respiration, where value of maximal respiration linked OCR is 100%. % basal oxygen consumption—parameter is expressed as a % of basal oxygen consumption, where value of basal oxygen consumption linked OCR is 100%. Figure 7. shows the linear relationship of AML 12 cell viability and (**a**) NM OCR, (**b**) NM OCR as % of maximal respiration, (**c**) NM OCR as % of basal oxygen consumption, (**d**) ATP OCR, (**e**) ATP OCR as % of maximal respiration, (**f**) ATP OCR as % basal oxygen consumption. Figure 7. shows the linear relationship of C2C12 cell viability and (**g**) NM OCR, (**h**) NM OCR as % of maximal respiration, (**i**) NM OCR as % of basal oxygen consumption, (**j**) ATP OCR, (**k**) ATP OCR as % of maximal respiration, (**l**) ATP OCR as % basal oxygen consumption. Figure 7. shows the linear relationship of NIT-1 cell viability and (**m**) NM OCR, (**n**) NM OCR as % of maximal respiration, (**o**) NM OCR as % of basal oxygen consumption, (**p**) ATP OCR, (**r**) ATP OCR as % of maximal respiration, (**s**) ATP OCR as % basal oxygen consumption.

To further investigate the link between non-mitochondrial OCR, ATP-production-linked OCR in normal conditions and cell viability under normal and hypoxic conditions, we calculated linear regressions, presented in Figure 7a–s. For both hepatic and muscle cells, the fraction of NM OCR in basal oxygen consumption seems to be the best predictor of cell survivability in different hypoxic conditions (Figure 7c,i). With increased NM OCR fraction in basal cellular respiration, cell viability decreases no matter the conditions. Furthermore, actual NM OCR in pmol/min does not seem to indicate cell survival (Figure 7a,g,m). As ETC is a significant producer of ROS in cells [52,53], the rise in NM OCR following the increased OXPHOS does not seem to have a predictable negative impact on cell survival.

The ATP-production-linked OCR expressed as a % of basal oxygen consumption seems to be the best predictor of cell survival (Figure 7f,l,s). Cell survival is positively impacted with an increase of ATP production fraction of OCR in hypoxic and normoxic conditions. For the C2C12 cell line, a similar relationship is visible for the ATP-production-linked OCR expressed as % maximal respiration, probably because of minor spare capacity observed within these cells. High ATP-production-linked OCR in normal conditions seems to predict higher survival when cells are exposed to hypoxia. However, it does not seem to be directly correlated with NIT-1 and AML 12 survival in a normoxic environment (Figure 7d,p). NIT-1 cells have a positive impact on viability from increased ATP production fraction of OCR only in normal and mild hypoxia but not in the higher hypoxia (Figure 7r,s).

## 3. Conclusions

The addition of deoxycholic acid overall increased the strength of gels, increasing surface tension and shear stress, with retained shear-thinning properties. This significantly impacted three cell lines (muscle, liver, and pancreatic beta cells) that were subjected to novel DCA-enhanced nanogel delivery matrices. Gels with stronger bonds lead to slightly lowered viability and decreased aerobic respiration, with a noticeable shift in cellular metabolism to glycolysis. Therefore, these hydro/nanogels may be more suitable for drug delivery than cell delivery matrices.

## 4. Materials and Methods

### 4.1. Materials

Deoxycholic acid (DCA), sodium alginate (SA, low viscosity) and Poloxamer 407 were acquired from Sigma Chemical Co. (St. Louis, MO, USA). The gel (water-soluble) was acquired from the Australian Medical Association (Perth, Australia). Dulbecco’s modified Eagle’s medium (DMEM), foetal bovine serum (FBS), and needed supplements were acquired from Sigma Chemical Co. (MO, USA).

### 4.2. Cell Culture

AML 12 was cultured in DMEM with 10% FBS and further supplementation based on published protocol [54]. C2C12 were grown in DMEM (10% FBS and 1% penicillin-streptomycin) following protocol for non-differentiated myotubes [55]. NIT-1 were grown in DMEM (supplemented with 10% FBS, 5.5 mmol glucose), Sigma-Aldrich, MO, USA), and free amino acids following a published protocol [56]. The cells were incubated in a humidified atmosphere of 5% CO_2_ at 37 °C. The media was changed every day for C2C12 and every other day for AML-12 and NIT-1.

### 4.3. Gel Preparation

Gels were prepared by mixing the water-soluble gel with sodium alginate (SA), chitosan, and DCA with deionised water under sterile conditions, following the formulations, as following. The water soluble gel (7%), SA (1.5%) and chitosan (0.3%) were used as appropriate, while DCA was used as (1.8, 2.2, and 2.4%) correspondingly, as described below.

For viability and hypoxia and seahorse assays, 20 µL of gel was added per well and left for 30 min under constant UV light. Freshly detached cells were added to gels and thoroughly mixed with a pipette. After mixing, the appropriate cell media was added. Each cell line was examined on a separate 96-well plate.

### 4.4. Rheology

A Visco-88 viscometer (Malvern Instruments, Malvern, UK) was utilised for viscosity, torque, shear stress, and shear rate measurements [56]. The Zeta potential was determent using a 3000HS Zetasizer (Malvern, UK). Surface tension was measured by a tensiometer (Sigma 703) [57]. All measurements were executed at room temperature.

### 4.5. Scanning Electron Microscopy (SEM)

Samples were freeze dried using Dynavac FD3 Freeze Dryer (Dynapumps, Seven Hills, Australia) for 48 h on −45 °C under vacuum. Afterwards, SEM (Zeiss Neon 40EsB FIBSEM, Carl Zeiss Microscopy GmbH, Jena, Germany) was utilised for surface morphology imaging [58].

### 4.6. Cell Viability Assay

A MTT (2,5-diphenyl-2,4,5-trimethylthiazol-2-yl) assay was used to observe the impact of nanogels on cell viability. Stock MTT solutions were prepared at 5 mg/mL within 24 h of use. All groups were treated in the same manner, including gel-free controls.

Following 24 h incubation, 20 µL of the stock MTT solution was added to each well containing cell-loaded nanogels or control wells. Resultant MTT-formazan was dissolved in 100 µL dimethyl sulfoxide (DMSO). The purple solution was then analysed via photometry at 550 nm in triplicates [59]. Obtained data were normalised and presented as a percentage, where 100% is the viability of cells in control wells.

### 4.7. Hypoxia Assays

Freshly detached cells were mixed with 80 µL of 50 µM or 100 µM solutions of CoCl_2_ in an appropriate cell media and left for 20 min before mixing them with gels. After mixing, a further 100 µL of the CoCl_2_-enriched media was added. After 24 h incubation, cell viability was assessed with the MTT assay [60].

### 4.8. Bioenergetic Parameters

After 24 h incubation, real-time mitochondrial activities were measured in hepatic, muscle, and pancreatic beta cells using a Seahorse Flux Analyser XF 96 (Agilent Technologies, Santa Clara, CA, USA) via an in-house-developed method [61,62].

### 4.9. Statistics

GraphPad Prism v9.3 (Graphpad, Inc., San Diego, CA, USA) for the data analysis, regressions, and one-way ANOVA/two-way ANOVA were conducted. Tukey HSD with post hoc comparisons of means was performed when the data were statistically significant. Data are shown as a mean ± SEM with *n* = 3, and the data were considered statistically significant at *p* < 0.01 (*) or *p* < 0.05 (**).

## Data Availability

The data presented in this study are available on request from the corresponding author. The data are not publicly available due to author property agreements.
