# Correction: Kovacevic et al. The Effect of Deoxycholic Acid on Chitosan-Enabled Matrices for Tissue Scaffolding and Injectable Nanogels. *Gels* 2022, *8*, 358

**DOI:** 10.3390/gels11121016

**Published:** 2025-12-18

**Authors:** Bozica Kovacevic, Corina Mihaela Ionescu, Melissa Jones, Susbin Raj Wagle, Michael Lewkowicz, Maja Đanić, Momir Mikov, Armin Mooranian, Hani Al-Salami

**Affiliations:** 1 The Biotechnology and Drug Development Research Laboratory, Curtin Medical School and Curtin Health Innovation Research Institute, Curtin University, Perth, WA 6102, Australia; bozica.kovacevic@postgrad.curtin.edu.au (B.K.); c.ionescu@postgrad.curtin.edu.au (C.M.I.); melissa.a.jones@postgrad.curtin.edu.au (M.J.); susbinraj.wagle@postgrad.curtin.edu.au (S.R.W.); mikhael.lewkowicz@graduate.curtin.edu.au (M.L.); 2 Hearing Therapeutics Department, Ear Science Institute Australia, Queen Elizabeth II Medical Centre, Perth, WA 6009, Australia; 3 Department of Pharmacology, Toxicology and Clinical Pharmacology, Faculty of Medicine, University of Novi Sad, 21101 Novi Sad, Serbia; maja.djanic@mf.uns.ac.rs (M.Đ.); momir.mikov@mf.uns.ac.rs (M.M.)

In Figure 1b [1], the unit “mPas” should be revised to “Pa·s.” In Figure 1d, the Torque unit “mN/M” should be revised to “mN·m.” For Figure 7 (legend and discussion), “basal respiration” should have been “basal oxygen consumption.” The correct Figure 1 and Figure 7 are as follows.

The authors state that the scientific conclusions are unaffected. This correction was approved by the Academic Editor. The original publication has also been updated.

## Figures and Tables

**Figure 1:**
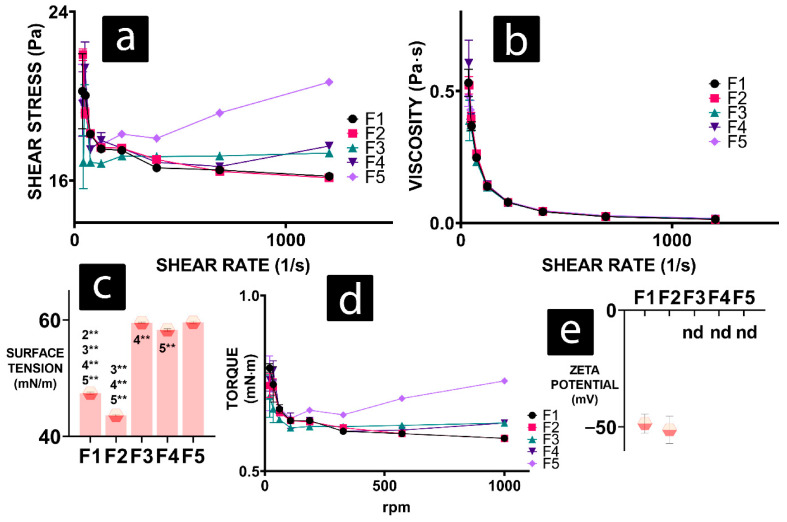
Rheology results of F1 to F5 formulations presented as values of (**a**) shear stress, (**b**) viscosity, (**c**) surface tension, (**d**) torque, and (**e**) zeta potential. *p* < 0.01 (*) or *p* < 0.05 (**).

**Figure 7:**
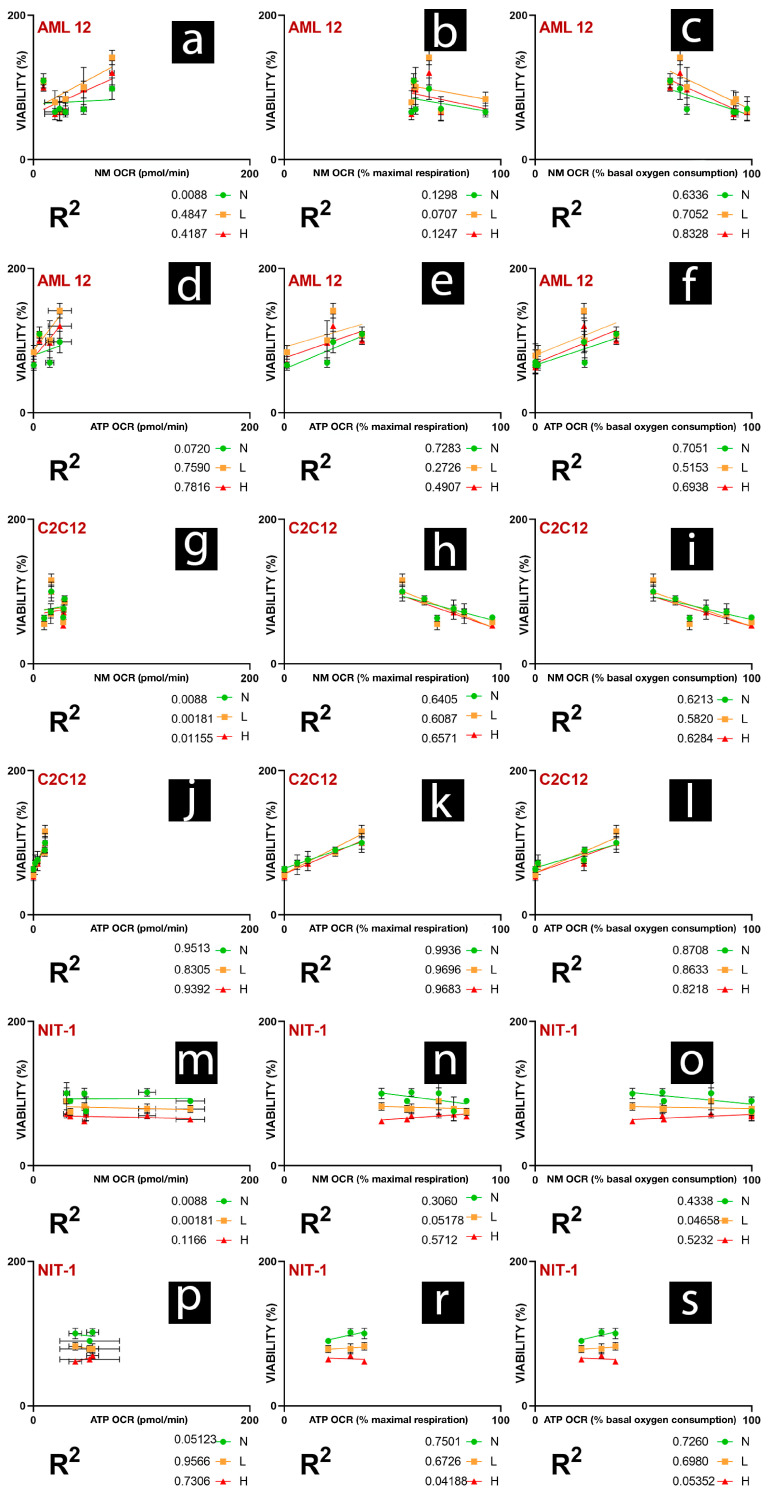
Relationships between bioenergetics in normoxic conditions and viability under normal, low, and high hypoxia. NM OCR, non-mitochondrial-linked OCR; ATP OCR, ATP-production-linked OCR. % maximal respiration—parameter is expressed as a % of maximal respiration, where value of maximal respiration linked OCR is 100%. % basal oxygen consumption—parameter is expressed as a % of basal oxygen consumption, where value of basal oxygen consumption linked OCR is 100%. Figure 7. shows the linear relationship of AML 12 cell viability and (**a**) NM OCR, (**b**) NM OCR as % of maximal respiration, (**c**) NM OCR as % of basal oxygen consumption, (**d**) ATP OCR, (**e**) ATP OCR as % of maximal respiration, (**f**) ATP OCR as % basal oxygen consumption. Figure 7. shows the linear relationship of C2C12 cell viability and (**g**) NM OCR, (**h**) NM OCR as % of maximal respiration, (**i**) NM OCR as % of basal oxygen consumption, (**j**) ATP OCR, (**k**) ATP OCR as % of maximal respiration, (**l**) ATP OCR as % basal oxygen consumption. Figure 7. shows the linear relationship of NIT-1 cell viability and (**m**) NM OCR, (**n**) NM OCR as % of maximal respiration, (**o**) NM OCR as % of basal oxygen consumption, (**p**) ATP OCR, (**r**) ATP OCR as % of maximal respiration, (**s**) ATP OCR as % basal oxygen consumption.
